# Using Lean Six Sigma to improve rates of day of surgery admission in a national thoracic surgery department

**DOI:** 10.1093/intqhc/mzz083

**Published:** 2019-12-22

**Authors:** Rachel Brown, Petra Grehan, Marie Brennan, Denise Carter, Aoife Brady, Eoin Moore, SeÁn Paul Teeling, Marie Ward, Donna Eaton

**Affiliations:** 1 Mater Misericordiae University Hospital, Eccles Street, Dublin 7, Ireland; 2 UCD Health Sciences Centre, University College Dublin, Belfield, Dublin 4, Ireland; 3 Centre for Innovative Human Systems, School of Psychology, Trinity College Dublin, the University of Dublin, Dublin 2, Ireland

**Keywords:** Lean Six Sigma, enhanced recovery after surgery, thoracic surgery

## Abstract

**Objective:**

The aim of this study is to improve rates of day of surgery admission (DOSA) for all suitable elective thoracic surgery patients.

**Design:**

Lean Six Sigma (LSS) methods were used to enable improvements to both the operational process and the organizational working of the department over a period of 19 months.

**Setting:**

A national thoracic surgery department in a large teaching hospital in Ireland.

**Participants:**

Thoracic surgery staff, patients and quality improvement staff at the hospital.

**Intervention(s):**

LSS methods were employed to identify and remove the non-value-add in the patient’s journey and achieve higher levels of DOSA. A pre-surgery checklist and Thoracic Planning Meeting were introduced to support a multidisciplinary approach to enhanced recovery after surgery (ERAS), reduce rework, improve list efficiency and optimize bed management.

**Main Outcome Measure(s):**

To achieve DOSA for all suitable elective thoracic surgery patients in line with the National Key Performance Indicator of 75%. A secondary outcome would be to further decrease overall length of stay by 1 day.

**Results:**

Over a 19 month period, DOSA has increased from 10 to 75%. Duplication of preoperative tests reduced from 83 to <2%. Staff and patient surveys show increased satisfaction and improved understanding of ERAS.

**Conclusions:**

Using LSS methods to improve both operational process efficiency and organizational clinical processes led to the successful achievement of increasing rates of DOSA in line with national targets.

## Introduction

Enhanced recovery after surgery (ERAS) is a multi-modal, evidence-based approach to delivering healthcare [1]. It is designed to optimize the surgical care pathway from point of referral to discharge [2]. The principles of ERAS have been applied to a variety of surgical specialities. Most recently, it has been adopted to thoracic surgery and considered to be the optimal “standard of care” for elective surgery [3].

**Figure 1:**
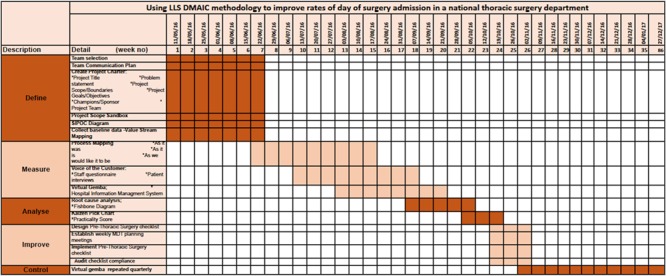
Gantt chart: research study timeline.

ERAS is designed to optimize the patient pathway. Benefits of ERAS include reduced perioperative stress response, reduction in complications, a faster return to baseline function [4], decrease in post-operative complications [5, 6] and reduction in length of stay (LOS) [7, 8]. Scarci *et al.* [6] and Lemanu *et al.* [9] further described significant cost savings following implementation of an ERAS programme for thoracic patients. It is reported that patients are better prepared for surgery if they are given a date for surgery; an admission time and pre-operative tests are completed at least 48 h in advance [10]. Day of surgery admission (DOSA) is an integral component of ERAS [10–12].

With the expansion of a National Thoracic Surgery Service from 114 cases in 2010 to 683 cases in 2017, the service demand required the implementation of a thoracic ERAS programme. To facilitate the introduction of the first ERAS programme in the Republic of Ireland, a Thoracic Surgery Advanced Nurse Practitioner (ANP) was employed to the service. Despite cost savings and a reduction in postoperative LOS, there remained multiple barriers to achieving DOSA.

The national key performance indicator (KPI) for DOSA is 75% for elective surgery cases. The majority of elective thoracic surgery patients were attending an established Pre-operative Assessment Clinic (POAC); however, DOSA was only achieved in 10.9% of patients in 2016. Despite attendance at the POAC, the percentage of patients having unnecessary repeated preoperative tests on admission to hospital was 67% in 2016.

Recognising the complexity involved in implementing a large perioperative quality improvement programme such as DOSA, the Lean Six Sigma (LSS) method of quality improvement was adopted. This has previously been adopted by other surgical services and is referenced in the NHS ERAS report as a suitable method for implementation [2].

Lean is a quality improvement methodology described ‘as a systematic approach to identifying and eliminating waste or non-value add activities in a process through continuous improvement [13, p1]’. Six Sigma is described as a data-driven process, the methodology of which is used to realize stable and predictable results, decreasing process variation and defects to a Six Sigma level [14].

Integration of the two concepts began in the 1990s and early 2000s, and the phrase ‘Lean Six Sigma’ was created. The five-stage improvement method of Define, Measure, Analyse, Improve and Control (DMAIC) adopts tools from both methodologies, therefore increasing both the speed and accuracy of the process improvement. The LSS DMAIC approach was utilized as the framework for this study and a number of LSS methods were applied.

The aim of this study was to improve rates of DOSA for all suitable elective thoracic surgery patients in line with the national KPI of 75% [15]. A secondary outcome would be to further decrease overall LOS by 1 day.

## Methods

### Study context

The hospital where the study was carried out is the national centre for cardiothoracic surgery and heart and lung transplantation in Ireland. It is a 600-bed teaching hospital providing a range of frontline and specialist services on a regional and national level. The thoracic surgery department was established in 1991 and provides treatment to patients with lung conditions such as cancer, fibrosis and emphysema. The first whole-time thoracic surgeon in Ireland was employed in the department in 2011; however, due to the exponential growth of the service in terms of both volume and complexity, there are now two whole-time thoracic surgeons employed with associated support staff. The department comprises 49 beds (shared with cardiac surgery programme) including a cardiothoracic high dependency unit and the national heart and lung transplant unit.

The hospital began to implement the LSS programme in 2013, and now projects are supported across the hospital by the Lean Academy. A project team was established in 2016 to support implementation of DOSA. The team was a multidisciplinary team (MDT) including a thoracic surgery ANP, a Cardiothoracic High Dependency Unit Clinical Nurse Manager (CNM) 2, Senior Physiotherapist, Patient Flow CNM, Radiology Administrator and an Infection Control Clinical Nurse Specialist. The team worked in conjunction with the thoracic surgeons to outline the scope of the study and to identify obtainable study goals. Weekly meetings were held over a 6-month period, and multiple LSS methods were applied, which will be outlined below using the structure of DMAIC.

Summary of research timetable by LSS phase:

Define: 11^th^ May 2016—22^nd^ June 2016.

Measure: 22^nd^ June 2016—21^st^ September 2016.

Analyse: 7^th^ September 2016—19^th^ October 2016.

Improve: 19^th^ October 2016—2^nd^ November 2016.

Control: January 2017—December 2017 (Fig. 1).

#### Define

In order to define the problem, the project team created a project charter which through using a project scope sandbox defined what was in and out of scope for the study.

What was considered to be in scope:

- Elective thoracic surgery patients that are suitable for DOSA, for example—video-assisted thoracoscopic surgery and lobectomies.
- Patients under the care of two named thoracic surgeons. These surgeons work solely with thoracic caseloads, which increased the potential pool of patients suitable for DOSA while ensuring that the sample size remained measurable and robust.

What was considered to be out of scope:

- Emergency admission thoracic patients. These patients often need to be scheduled in an emergency capacity which prohibited their access to pre-operative assessment, therefore deeming them unsuitable for DOSA.
- All other patients.

Early identification of all relevant stakeholders was facilitated through a Suppliers, Inputs, Process, Outputs and Customers (SIPOC) diagram. LSS methods place a strong emphasis on team-building tools, including the SIPOC, that help the team collaborate, engage and negotiate with a variety of disciplines. This provokes greater buy-in from staff to the initiative and helps to create sustainable change.

The LSS method of value stream mapping (VSM) was employed to create an overview of the current ‘AS IS’ thoracic surgery patient journey. VSM highlights non-value add along the patients’ journey [16]. Scoville and Little [17] describe VSMs as detailed steps in a process, along with the related information flow and data, to quantify waste, cycle time and other process characteristics.

Patient data was retrospectively reviewed from an electronic hospital system. This data was used to create a basic process map. All of the stakeholders identified in the SIPOC were then asked to review and refine this map given their role and understanding of the process. It was agreed that for this study, the core VSM is the patient’s journey from the first out-patient appointment with the surgeon to when they reach the theatre. Before the implementation of ERAS in the hospital, a process map had identified 42 steps in the patient journey. With the implementation of ERAS in 2016, the number of steps in the process had decreased to 36. However, there was still a large amount of non-value added steps identified including not attaining DOSA. Much of this was recognized to be related to the admission process for this population. Wackerbarth *et al.* [18] identified a key success factor in LSS as understanding and focusing the scope of the study.

#### Measure

This stage involved gathering more detailed data on both the current ‘AS IS’ process as well as measuring what LSS refers to as the voice of the customer (VOC) of the process. For this, the thoughts and opinions from both patients and nursing staff were gathered in relation to the process. VOC was gathered through anonymous questionnaires administered to nursing staff. Nursing staff in the cardiothoracic ward were invited to participate in the study. This group was chosen as they were considered pivotal to the successful implementation of DOSA. They were also one of the stakeholder groups which would be most impacted by its implementation. In 2016 there was a response rate of 57.5% (*n* = 23), and in 2017 there was a response rate of 55% (*n* = 22). Participation was voluntary and all completed questions were anonymized.

**Figure 2:**
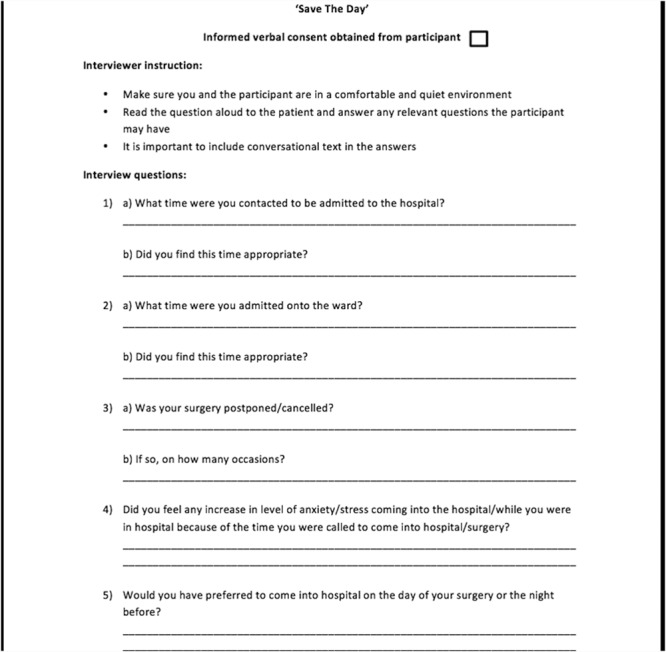
Sample patient questionnaire.

Face-to-face interviews with post-operative patients were carried out in the post-thoracic surgery clinic two weeks post-operatively. Five patients were randomly selected on the day. A treatment room in a quiet area was provided away from the waiting area to ensure privacy and confidentiality for the patients. These interviews were conducted by two members of the project team not working in the clinical area to ensure that a conflict of interest did not occur. Patients were asked eight questions in order to obtain both qualitative and quantitative data (Fig. 2).

Data was also collected using Gembas or “walking the process” as described by Womack [19]. Data was gathered on the patient journey using the hospital computer system ‘Patient Centre’. This captured data including surgeon, procedure, attendance at POAC and DOSA confirmed surgical date given and whether pre-operative tests had been completed, allowing evaluation of repeat testing/re-work^1^ and DOSA.

#### Analyse

**Figure 3:**
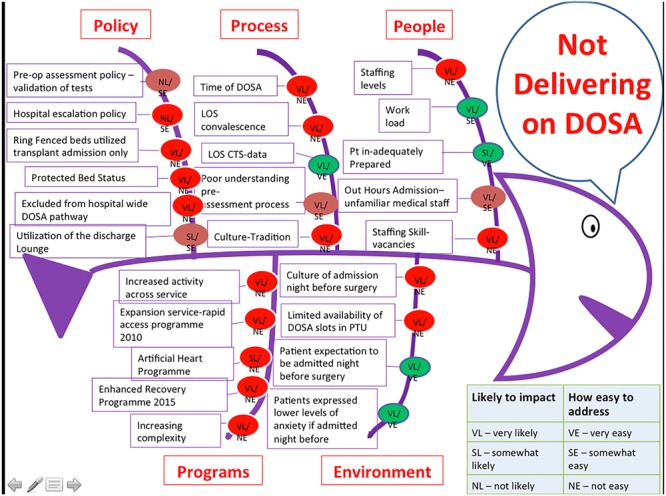
Fishbone diagram of causes of non-value add in the process.

The data from the VSM, Gemba and VOC were analysed. The group used a fishbone diagram (Fig. 3) to carry out a root cause analysis (RCA) on the non-value add identified from these methods [16].

This highlighted a number of issues with the current process which included:

- An excess number of steps in the patient pathway
- The large volume of rework that was taking place and the associated costs to the patient and hospital
- Other issues which can be seen in Fig. 3.

Using a Kaizen Pick chart, DOSA was identified as having a big payoff if implemented but also had a high level of difficulty to implement given the historical cultural practice of bringing patients into the hospital the night before surgery. However, the reduction in pre-operative testing was noted to have a smaller payoff but potentially an easier level of implementation. In an effort to reduce the costly rework and facilitate DOSA, the LSS project team designed a ‘pre-thoracic surgery checklist’. This was designed to formally document which tests had been completed for the patient and at what stage, highlighting if any further investigations were required.

#### Improve

The intervention consisted of the following components:

- Development and implementation of the pre-thoracic surgery checklist
- The establishment of a weekly planning meeting for the thoracic surgery MDT
- A weekly audit of the pre-thoracic surgery checklist

A weekly thoracic surgery MDT planning meeting was established. This required the commitment of all the team to attend. This included the thoracic consultants, surgical team, thoracic ANP, CNMs from the ward and theatre, senior physiotherapist and other members of the MDT on a case by case basis. Buy-in to attend was achieved through presenting the findings from the analysis of the data at a grand round on DOSA and through colleague-to-colleague invitation where the benefits of the study where outlined.

The pre-thoracic surgery checklist was introduced to this weekly MDT meeting where it formed part of the discussion on the patient and was stapled to the patients chart to provide an easy checklist for the admitting team.

**Figure 4:**
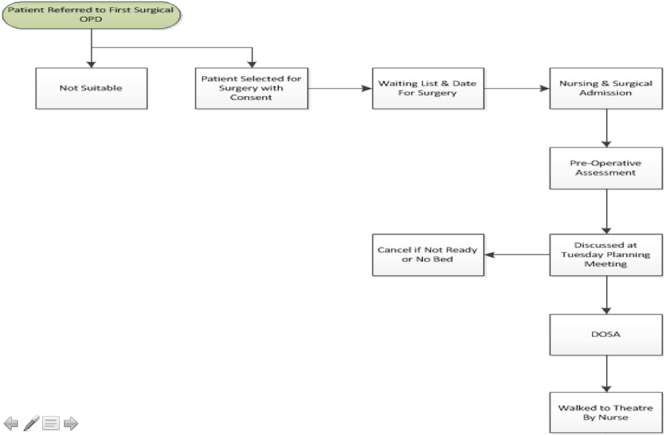
Streamlined process map for 2017 with the introduction of DOSA.

The process mapping exercise initially identified 36 steps in the patients’ journey. Through the application of LSS methodology, this was reduced to 10 steps. This greatly streamlined the process for thoracic surgery patients, and the final process map is shown in Fig. 4.

**Figure 5:**
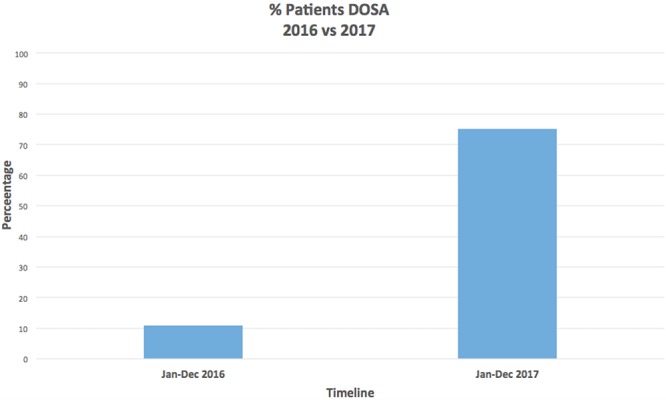
Percentage DOSA for patients.

**Figure 6:**
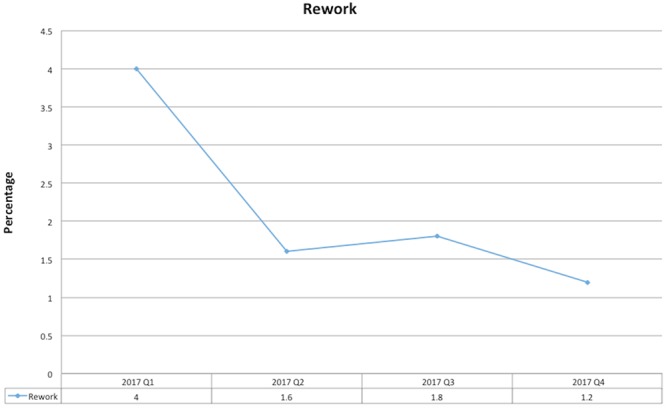
The percentage of patients in 2017 having unnecessary re-work of pre-operative tests at each quarterly analysis.

#### Control

To evaluate sustainability of the process, improvement outcomes for LOS, DOSA and re-work were measured at quarterly intervals over a period of 12 months from January to December 2017. These data were audited using the virtual Gemba process to facilitate timely and accurate recording.

## Results

### Study outcomes

#### DOSA

DOSA for elective thoracic surgery patients was 10.9% in 2016, and following the introduction of the DOSA pathway, this increased to 75.3% in the year 2017. Please see Fig. 5.

The patient interviews highlighted that patients were satisfied with the idea of DOSA in 2016. In 2017 with the implementation of DOSA the patient satisfaction questionnaire was repeated. Patients were satisfied with DOSA and no patient reported increased anxiety or stress related to DOSA. All patients in 2017 were satisfied with the notice given prior to admission. In 2016, 57% of nurses completed the staff questionnaire compared with 56% in 2017. The nursing questionnaire asked staff if they felt that DOSA was important and whether they felt the hospital was addressing this need. Of the respondents in 2016, most felt that DOSA was important but only 7% felt that the need was being met. This improved in 2017 when DOSA had been introduced and 57% felt the need was now being met.

#### Duplication of preoperative test ‘rework’

The data collected from the initial virtual Gemba showed that 83% of patients who had attended POAC (and were deemed ready for DOSA) had rework carried out on admission despite this not being required by the POAC. In 2016, this decreased to 67% principally through staff education. With the implementation of this study, this rate dramatically decreased to only 2% of patients. Figure 6 illustrates the quarterly data for 2017 and how this success was sustained into the control phase.

#### LOS

The post-operative LOS decreased from 4.2 to 3.9 days during the control phase, January to December 2017 with over 75% of elective patients also achieving DOSA (Table 1).

**Table 1:** Study outcome summary table

	Outcome(s)	Achieved by
1	Increased DOSA from 10.9 to 75.3% from 2016 to 2017	LSS DMAIC framework
2	Reduction in duplication of rework (blood work, chest X-rays) from 67 (2016) to <2% (2017)	Designing and implementing a pre-thoracic surgery checklist Implementing a weekly thoracic MDT meeting
3	LOS	LSS DMAIC framework

## Discussion

ERAS has been shown to facilitate a cohesive, streamlined approach to elective surgery yielding significant saving in terms of hospital bed days [2, 6] and improved patient satisfaction [6, 12, 20, 21].

The Republic of Ireland’s first Thoracic ERAS programme was implemented in 2015 in this hospital by the thoracic surgery ANP; however, despite implementing a robust ERAS programme, DOSA was not achieved. Through the use of the LSS tools, DOSA was achieved in over 75% of elective patients in 2017. The success of this study was in part due to the diverse team of professionals working on the study who were all undertaking the LSS Green Belt programme in the hospital. The majority of team members had limited experience working in the thoracic surgery department prior to this project. The team worked cohesively in a structured and goal-orientated manner over the designated 6-month period using the LSS tools. In addition to a framework for improvement, LSS training provides a set of team-building tools that help individuals to collaborate, engage and negotiate with a variety of disciplines across the organization in pursuit of change [22].

While change implementation can be challenging, the team found that relevant stakeholders were highly engaged in this process as the demand for this project was well recognized and supported within the thoracic team. Aligning individual projects to the strategic goals of the organization and recognising the voices of all the customers affected by the project is essential to sustaining LSS [22].

Guidance and support from the LSS Black Belt supervisor greatly aided the success of change implementation in this study. This was provided by both formal and informal support which allowed for the study to blend with daily roles and responsibilities of the senior members of the team.

A key factor to the success of this study was the engagement from all stakeholders and in particular the buy-in from the medical professionals, with the study sponsor being a thoracic consultant. These factors were also identified as critical success factors by Proudlove *et al.* [23].

Admission of patients the day before surgery has been historical in this service, and therefore implementing DOSA changes were initially received cautiously by those working in the service. However, this study demonstrates that with an effective study design and robust data collection, change for the betterment of the hospital, the staff and most importantly the patient population is possible. The application of LSS for healthcare is well-documented in the literature, where studies report improvement in clinical processes, identification and elimination of waste from patient pathways, empowering and enabling staff to examine their own workplace increasing quality, safety and efficiencies [24–26].

Early in the define phase, the problem identified from our initial scoping exercise was the failure to achieve DOSA which is an integral part of the ERAS programme. The use of LSS tools allowed the team to identify this early and to narrow the focus of the study. Only a small proportion of the surgical patients from the first visit in outpatients to the door of theatre were included. A failure to narrow the scope of the study is one of the challenges encountered by LSS teams when implementing change [23].

It is vital to gather information from all sources during the measurement phase. By using the interview techniques for patients, the team was able to establish what was working well at present, e.g. spacious ensuite single rooms, and what was not working, e.g. operation cancellations. This helped to reaffirm these ideas within the team. The questionnaire technique for nursing staff allowed the team to gather information from a larger cohort with diverse perspectives and priorities. Priorities such as decreasing re-work and improving communication between the medical team and nursing staff were then addressed in the implementation phase by the establishment of the weekly MDT and the pre-thoracic surgery checklist.

## Limitations

Time limitations were a challenge for all group members and not readily anticipated from the outset. It was increasingly difficult to take time from clinical hours to preform Gemba assessments and patient interviews as all team members were senior staff in their areas of expertise. The sample size of patient interviews was low (*n* = 5), and there was a lack of repeat VOC measurement undertaken for both patients and staff in the control phase.

Challenging LSS methods included the Actual Gemba, where team members followed the patient’s pathway on the ward which did not allow for sufficient data collection, so switching to retrospective data collection where team members followed the patient pathway electronically provided the team with more relevant data. The use of LSS tools enabled the study to be successful; however, a learning for the team was that the initial tools chosen may be unsuccessful in providing data, and therefore, other tools may need to be utilized to obtain the data required.

## Conclusion

Through the use of the LSS approaches and methods, DOSA was achieved in over 75% of elective patients in 2017. The success of this study was in part due to the diverse team of professionals working on the study. All six team members were employees of the organization where the project was implemented. This enabled face-to-face meetings with the group as required providing structure and support to the group, effective communication and any problems encountered to be teased out quickly enabling the stages of the study to move forward in a timely manner. Due to the varied backgrounds, each team member was able to draw on their own strengths and knowledge providing the group with a different perspective and opinions driving the study forward. Effective communication between the project team and the Thoracic MDT at the weekly planning meetings provided structure once implemented.

Having clear goals and objectives and identifying what is in and out of scope early on were vital. Early involvement and support from the relevant stakeholders are imperative; if they do not believe in the study, they will not be as invested in achieving the end goals. This can help overcome cultural barriers to change. This is where the support from stakeholders is vital as they will help drive the study forward as they share a common goal with the group to successfully implement the change.

## Footnotes

1 Re-work includes preoperative testing such as chest x-ray, blood sample analysis and microbiology screening.
