# Corrigendum to: Using Lean Six Sigma to improve rates of day of surgery admission in a national thoracic surgery department

**DOI:** 10.1093/intqhc/mzaa003

**Published:** 2020-03-09

**Authors:** Rachel Brown, Petra Grehan, Marie Brennan, Denise Carter, Aoife Brady, Eoin Moore, SeÁn Paul Teeling, Marie Ward, Donna Eaton

This manuscript has been amended to correct an author’s name. Denise Carter was initially incorrectly named in the author list as Donna Carter. This has now been corrected. Apologies for any confusion caused.

